# Biological variation of immunological blood biomarkers in healthy individuals and quality goals for biomarker tests

**DOI:** 10.1186/s12865-019-0313-0

**Published:** 2019-09-14

**Authors:** Najib Aziz, Roger Detels, Joshua J. Quint, David Gjertson, Timothy Ryner, Anthony W. Butch

**Affiliations:** 1 Department of Epidemiology, Fielding School of Public Health at University California Los Angeles (UCLA), 650 Charles E. Young Dr. South, Los Angeles, CA 90095-1772 USA; 2 David Geffen School of Medicine, UCLA, Los Angeles, California 90095 USA; 3 Department of Biostatics, Fielding School of Public Health, UCLA, Los Angeles, California 90095-1772 USA; 4 Department of Intercollegiate Athletes, UCLA, Los Angeles, California 90095-1772 USA

**Keywords:** Chemokine, Cytokines, Immune activation, Interleukin, Soluble markers

## Abstract

**Background:**

Cytokines, chemokines, adipocytokines, soluble cell receptors, and immune activation markers play an important role in immune responsiveness and can provide prognostic value since they reflect underlying conditions and disease states. This study was undertaken to investigate the components of biological variation for various laboratory tests of blood immunological biomarkers.

**Results:**

Estimates of intra-individual coefficient of variation (CV_I_) and inter-individual coefficient of variation (CV_G_) were examined for blood immunological biomarkers. Biomarkers with CV_I_ < 10% for both genders were CD3, CD4, and CD8 T-cells, serum levels of soluble cluster of differentiation 14 (sCD14), sCD163, and soluble glycoprotein 130 (sgp130). The CV_I_ for serum levels of adiponectin, interleukin-1 receptor antagonist (IL-1Ra), macrophage inflammatory protein 1 beta (MIP-1β), soluble CD40 Ligand (sCD40L), soluble interleukin-2 receptor alpha (sIL-2Rα), soluble interleukin-6 receptor (sIL-6R), soluble tumor necrosis factor receptor II (sTNF-RII), and tumor necrosis factor alpha (TNF-α) were between 11 and 20%. Biomarkers with CV_G_ < 20% were CD3 T-cell, and serum concentrations of sCD14, sCD40L, and sgp130. The biomarkers with CV_G_ > 40% were adiponectin, IL-1ra, leptin, MIP-1β, sCD163, and sIL-2Rα.

**Conclusion:**

The biological variations of biomarkers have important monitoring value for longitudinal investigation and are essential for quality specification of tests that are performed in the laboratory. The CV_I_ was relatively small while CV_G_ was comparatively large and mean values of each biomarker vary between subjects. The individuality of biomarkers significantly influences reference interval values. A majority of the biomarkers in this study had strong individuality and the result of each biomarker should be cautiously interpreted if using established reference interval values. Comparison of a patient’s test result with previous ones may be more useful than the usage of conventional reference values.

**Electronic supplementary material:**

The online version of this article (10.1186/s12865-019-0313-0) contains supplementary material, which is available to authorized users.

## Background

Immunological biomarkers such as serum cytokines, chemokines, adipocytokines, soluble forms of cell receptors, and immune activation markers can serve as surrogate markers for cellular activation and play an important role in the function of the immune system. Complex interactions between immune cells of the innate and adaptive immune systems are modified by the release of a variety of cell mediators that trigger inflammatory responses which help to eliminate and destroy foreign antigens. They serve numerous functions within our immune system and interact with specific cell types that correspond with different stages of disease.

Changes in the levels of these biomarkers along with changes in lymphocyte subset activity can provide important prognostic value by reflecting underlying disease conditions [1–7]. Thus, it is essential to be able to detect laboratory and clinically relevant changes by accurately measuring those blood biomarkers, making them potentially beneficial for the monitoring, diagnosis, and follow up of various diseases.

Clinical usefulness of these biomarkers depends upon: 1) their ability to account for a significant portion of the disease being evaluated; 2) the ability to be accurately, reproducibly, and reliably measured; and 3) the availability of the assay for widespread application [8]. Since blood levels of immunological biomarkers differ widely between individuals based on their gender, age, and other factors, baseline concentrations of biomarkers should be established for healthy individuals. In addition, the suitable biomarkers should have a low temporal intra-individual variability in healthy subjects otherwise any significant changes might not necessarily reflect disease processes.

Biological variation (BV) is often the most important source of variation over time for certain biomarkers and marked changes can occur during the neonatal, childhood, puberty, menopause, and aging process. In addition, certain biomarkers have biological rhythms that can vary diurnally, monthly, or seasonally [9].

There are a limited numbers of studies that have examined the BV of immunological biomarkers and lymphocyte phenotype and a majority of published studies have only investigated the BV associated with clinical chemistry and hematological biomarkers [10].

In this study we evaluated the short term (six weeks) temporal intra-individual variation (CV_I_) and inter-individual variation (CV_G_) of commonly requested laboratory tests for immunological biomarkers of serum/plasma **cytokines** such as interleukin-1 beta (IL-1β), interleukin-6 (IL-6), interleukin-12p70 (IL-12p70), interleukin-1 receptor antagonist (IL-1Ra), interferon gamma (IFN-γ), and tumor necrosis alpha (TNF-α); **chemokines** such as interleukin-8 (IL-8), macrophage inflammatory protein 1 alpha (MIP-1α) or CCL3, macrophage inflammatory protein 1 beta (MIP-1β) or CCL4, and regulated upon activation normal T cell expressed and secreted (RANTES) or CCL5; **adipocytokines** such as adiponectin and leptin; blood **soluble forms of cell surface receptors** such as soluble cluster of differentiation (CD)14 (sCD14), soluble CD25 (sCD25) or IL-2Rα, soluble CD40 Ligand (sCD40L), soluble CD120b (sCD120b) or sTNF-RII, soluble CD126 (sCD126) or sIL-6R, soluble CD130 (sCD130) or sgp130, and soluble CD163 (sCD163); **immune activation markers** such as neopterin and T and B lymphocyte phenotype. The clinical and research significance for each biomarker is outlined in the Additional file 4.

The aim of our study was to gather more information about the CV_I_ and CV_G_ of the biomarkers in healthy individuals and influences of CV_I_ and CV_G_ on the population based reference values as well as setting quality specification goals for precision, bias, and total error allowable of laboratory tests for those markers.

## Results

The mean serum concentration of five groups (cytokines, chemokines, adipocytokines, soluble cell receptors, and immune activation markers) of biomarkers and the mean percentage of each lymphocyte subset for twelve normal participants, along with coefficients of variation for analytical (CV_A_), intra-individual (CV_I_), and inter-individual (CV_G_) for six women, six men, and both genders are shown in Tables 1 and 2. Measured serum levels of IL-12p70 and MIP-1α for all twelve participants were lower than the minimum detectable dose.

**Table 1:** Mean value of biomarkers, analytical (CV_A_), intra-individual (CV_I_), and inter-individual (CV_G_) components of variation for eighteen serum biomarkers

		*Weighted*		*CV*_*I*_ *(%)*			*CV*_*G*_ *(%)*		
	*Biomarker (unit)*	*Mean*	*CV*_*A*_ *(%)*	Male	Female	Both	Male	Female	Both
Group I	IL-1β (pg/mL)	0.157	4.0^a^	38.0	22.0	30.9	NC^b^	42.0	27.0
	IL-6 (pg/mL)	0.961	6.4	23.0	20.0	21.5	33.0	19.0	26.4
	IL-1Ra (pg/mL)	419.0	6.0	15.0	17.3	17.0	42.0	37.7	43.0
	IFN-γ (U/L)	278	13.5	29.0	34.0	31.0	10.8	33.0	22.0
	TNF-α (pg/ml)	12.63	8.2	7.9	14.2	12.7	24.3	31.7	27.2
Group II	IL-8 (pg/mL)	12.16	3.5^a^	24.0	61.6	51.4	32.0	28.0	30.2
	MIP-1β (pg/mL)	101.0	4.5	18.0	22.0	20.0	22.0	82.0	57.0
	RANTES (ng/mL))	32.71	3.2	27.0	23.0	24.0	51.0	30.0	39.0
Group III	Adiponectin (μg/mL)	6.29	3.7	12.0	17.0	15.9	49.0	42.0	48.1
	Leptin (ng/mL)	7.66	7.1	18.0	31.0	31.0	66.0	50.0	68.0
Group IV	sCD14 (ng/mL)	1435.0	2.8	8.8	7.6	8.0	16.0	6.1	11.4
	sCD40L (ng/mL)	6.02	3.0	14.5	12.7	13.2	18.4	12.8	14.6
	sCD163 (ng/mL)	543.0	9.5	6.8	6.5	9.0	64.9	50.2	53.8
	sgp130 (ng/mL)	281.0	4.8	4.4	7.5	7.0	12.5	10.4	15.0
	sIL-2Rα (pg/mL)	796.0	3.6	9.0	19.0	15.1	34.0	49.0	41.7
	sIL-6R (ng/mL)	31.77	3.0^a^	18.2	12.9	16.3	18.5	18.9	20.3
	sTNF-RII (ng/mL)	2.17	10.1	17.6	15.8	17.4	36.0	25.9	28.9
Group V	Neopterin (nmol/L)	5.20	6.4	14.5	64.0	35.0	69.4	14.0	37.0

^a^: CV_A_ calculated from 5 duplicated and two level assay control samples, ^b^: not calculable (NC)

**Table 2:** Mean value lymphocyte phenotype, analytical (CV_A_), intra-individual (CV_I_), and inter-individual (CV_G_) components of variation for five lymphocyte phenotypes

*Biomarker*	*Weighted Mean (%)*	*CV*_*A*_ *(%)*	Intra-subject variation *CV*_*I*_ *(%)*			Inter-subject variation *CV*_*G*_ *(%)*		
Phenotype			Male	Female	Both	Male	Female	Both
CD3 (T cells)	73	1.0	3.0	4.1	3.6	8.3	13.6	10.8
CD4	46	3.2	6.8	5.6	6.1	10.5	20.9	20.7
CD8	25	2.4	6.9	5.7	6.7	20.3	43.6	35.5
CD19 (B cells)	12	4.1	22.0	12.6	17.4	27.4	37.0	31.9
CD56/16 (NK cells)	13	5.8	21.4	24.9	23.0	23.4	50.9	37.1

CV_A_ is analytical variation (mean CV of daily QC run for 20 days) expressed as a %CV

Biological variation (BV) is expressed as a percentage and intra-individual variation was relatively small while inter-individual was relatively large. Biomarkers with a CV_I_ < 10% for both genders were the percentage of CD3^+^, CD4^+^, and CD8^+^ T-cells; and serum concentrations of sCD14, sgp130, and sCD163. The CV_I_ for IL-1Ra, TNF-α, MIP-1β, adiponectin, sIL2Rα, sCD40L, sTNF-RII, and sIL6R ranged from 11 to 20%.

Biomarkers with a CV_G_ < 20% were the percentage of CD3^+^ T-cells and serum concentrations of sCD14, sCD40L, and sgp130. Biomarkers with the most inter-individual variability (CV_G_ > 40%) were serum concentrations of IL-1Ra, MIP-1β, adiponectin, leptin, sIL2Rα, and sCD163.

**Fig. 1:**
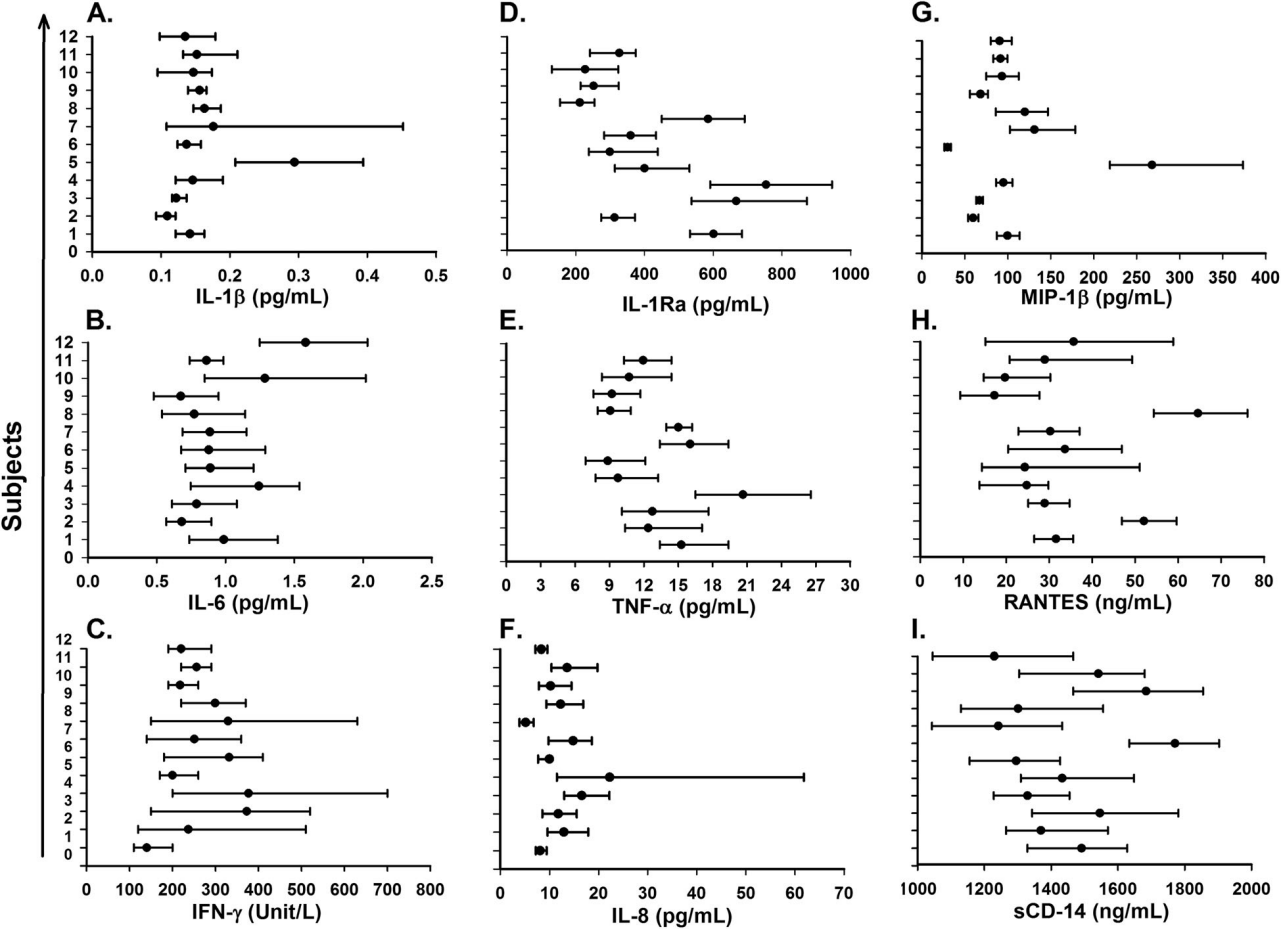
Mean (filled circle) and absolute range (error bar) for each subject (n=12) over six weeks, for serum level of IL-1β (**a**), IL-6 (**b**), IFN-γ (**c**), IL-1Ra (**d**), TNF-α (**e**), IL-8 (**f**), MIP-1β (**g**), RANTES (**h**), and sCD14 (**i**)

**Fig. 2:**
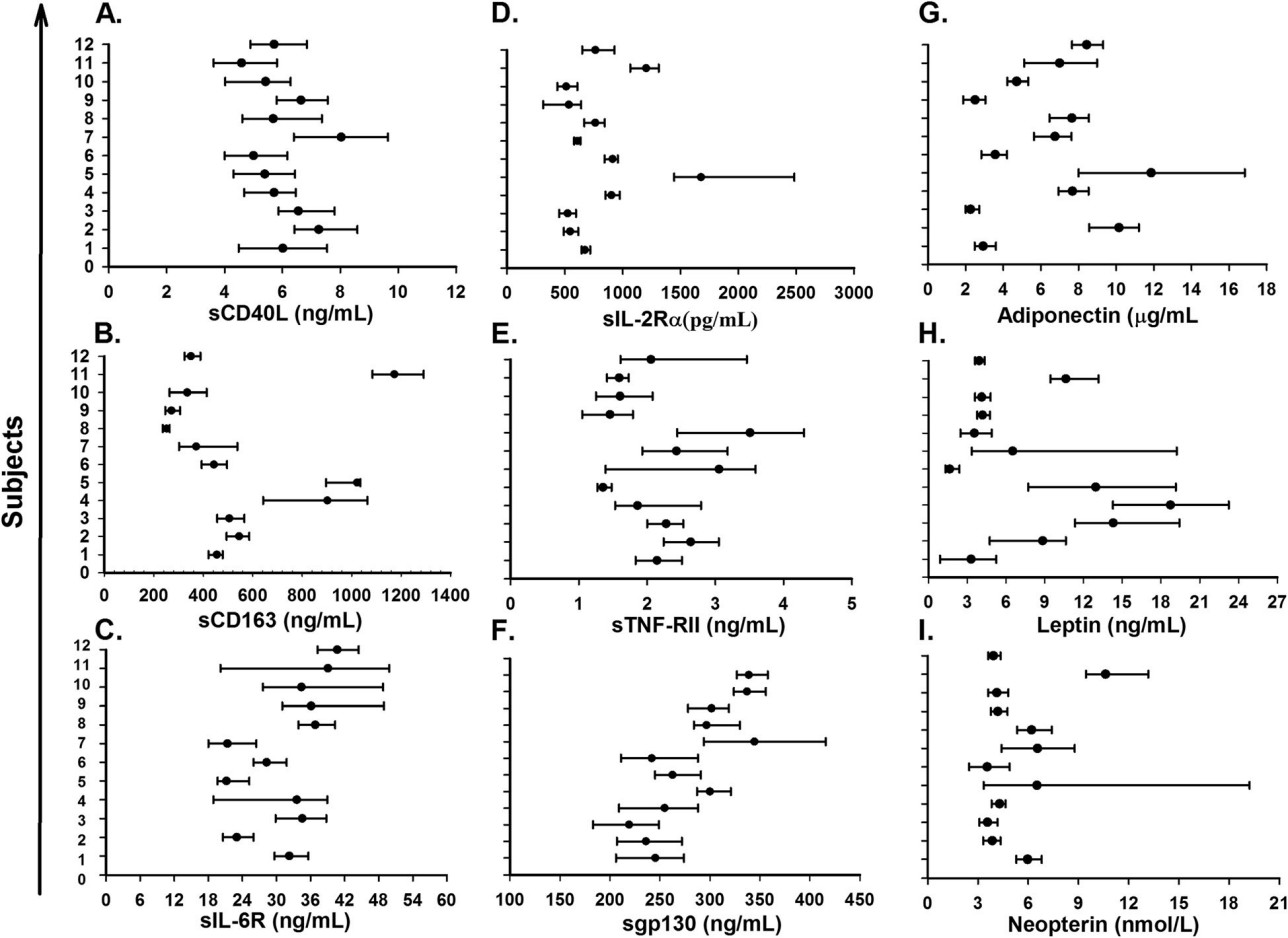
Mean (fialled circle) and absolute range (error bar) for each subject (n=12) over six weeks, for serum level of sCD40L (**a**), sCD163 (**b**), sIL-6R (**c**), sIL-2Rα (**d**), sTNF-RII (**e**), sgp130 (**f**), Adiponectin (**g**), Leptin (**h**), and Neopterin (**i**)

**Fig. 3:**
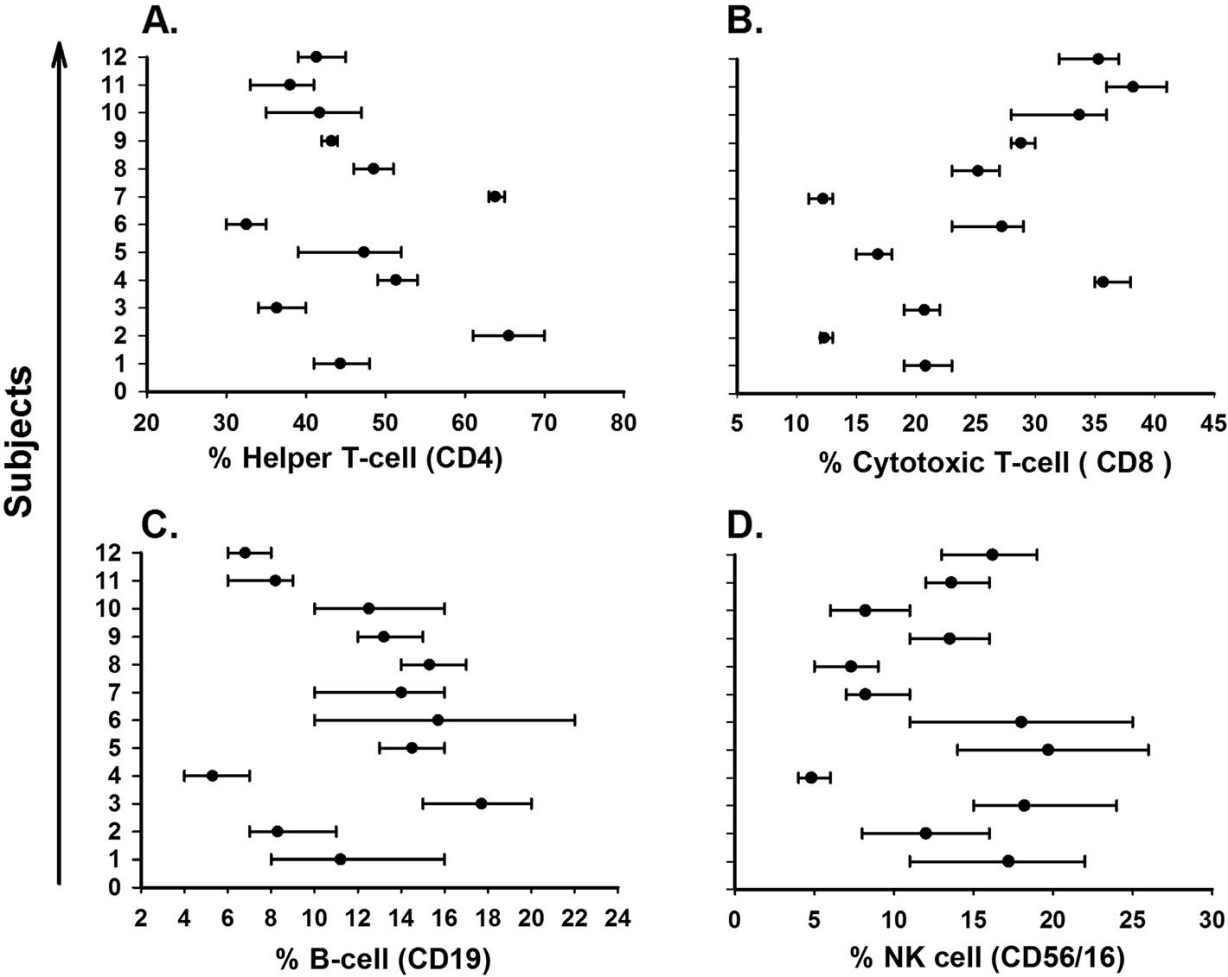
Mean (filled circle) and percentage range (error bar) for each subject (n=12) over six weeks, for helper T-cell (**a**), Cytotoxic T-cell (**b**), B-cell (**c**), and NK cell (**d**)

Women had higher CV_I_ for 10 out of 18 serum biomarkers compared to men, while men had a higher CV_G_ for 13 of 18 serum biomarkers. CV_G_ was not calculable in men for IL-1β, because the mean square of inter-individual was smaller than inter-individual (Table 1). The influence of biomarker individuality on conventional population reference values and interpretation of laboratory test results visualized by the mean and absolute range of each biomarker for the twelve healthy subjects is presented in Fig. 1 for IL-1β (A), IL-6 (B), IFN-γ (C), IL-1Ra (D), TNF-α (E), IL-8 (F), MIP-1β (G), RANTES (H), and sCD14 (I); in Fig. 2 for sCD40L (A), sCD163 (B), sIL-6R (C), sIL-2Rα (D), sTNF-RII (E), sgp130 (F), Adiponectin (G), Leptin (H), and neopterin (I); and in Fig. 3 for CD4 (A), CD8 (B), CD19 (C), and CD56/16 (D).

The *desirable* quality specifications for imprecision and bias (%), total error allowable at 95 and 99%, and the indices of individuality and heterogeneity for each of the biomarkers based on biological variation are calculated and summarized in Tables 3 and 4.

**Table 3:** Analytical goals for imprecision, bias, total error allowable (TE_a_) for *p* < 0.01 and *p* < 0.05, reference change value (RCV), Reliability coefficient (R), indices of individuality (II), and index of heterogeneity (IH) for eighteen serum biomarkers

	*Biomarker*	*Analytical goals for precision (%)*	*bias (%)*	*TE*_*a*_ *(%)*		RCV(%)	R	II	IH
				*p < 0.01*	*p < 0.05*	95			
Group I	IL-1β	15.4	10.1	35.5	46.0	95.6	0.38	1.14	0.49
	IL-6	10.6	8.5	26.2	33.6	66.6	0.55	0.85	0.35
	IL-1Ra	8.5	11.6	25.6	31.4	52.7	0.84	0.42	0.28
	IFN-γ	15.5	9.5	35.1	45.6	96.0	0.29	1.52	0.53
	TNF-α	6.3	7.5	17.9	22.2	39.3	0.79	0.55	0.24
Group II	IL-8 (CXCL8)	25.7	14.9	57.3	74.8	159.2	0.22	1.70	0.81
	MIP-1β (CCL4)	10.0	15.1	31.6	38.4	61.9	0.87	0.36	0.32
	RANTES (CCL5)	12.0	11.4	31.2	39.4	74.3	0.68	0.62	0.38
Group III	Adiponectin	7.95	12.7	25.8	31.2	45.2	0.90	0.34	0.26
	Leptin	15.5	18.7	44.3	54.8	96.0	0.79	0.47	0.50
Group IV	sCD14	4.0	3.5	10.1	12.8	24.8	0.62	0.74	0.13
	sCD40L	4.7	5.2	12.9	16.1	38.8	0.52	0.54	0.21
	sCD163	4.5	13.6	21.1	24.1	27.9	0.97	0.24	0.21
	sgp130	3.5	4.1	9.9	12.3	21.7	0.79	0.57	0.13
	sIL-2Rα	7.55	11.1	23.5	28.7	46.8	0.86	0.37	0.24
	sIL-6R	8.16	6.5	20.0	25.5	50.5	0.55	0.81	0.26
	sTNF-RII	8.7	8.4	22.8	28.7	53.9	0.69	0.70	0.32
Group V	Neopterin	17.5	12.7	41.6	53.5	108.4	0.47	0.96	0.56

Group I represents cytokines, Group II is chemokines, group III is adipocytokines, group IV represents soluble cell receptors, and group V is immune activation markers

**Table 4:** Analytical goals for imprecision, bias, total error allowable (TE_a_) for *p* < 0.01 and *p* < 0.05, reference change value (RCV), Reliability coefficient (R), index of individuality (II), and index of heterogeneity (IH) for five lymphocyte phenotypes

*Biomarker Phenotype*	*Analytical goal for precision (%)*	*bias (%)*	*TE*_*a*_ *(%)*		*RCV (%)*	R	II	IH
			*p < 0.05*	*p < 0.01*	*P < 0.05*			
CD3 (T cells)	1.8	2.8	5.8	7.0	11.1	0.89	0.34	0.06
CD4	3.1	5.4	10.5	12.6	19.2	0.90	0.33	0.11
CD8	3.4	9.0	14.6	16.8	19.8	0.96	0.20	0.11
CD19 (B	8.7	9.1	23.4	29.4	49.6	0.76	0.56	0.28
cells) CD56/16 (NK cells)	11.5	10.9	29.9	37.7	65.7	0.71	0.64	0.37

In addition, the mean, median, and inter–quartile range (IQR) of the biomarkers were calculated for six male, six female, and both genders, and presented in Additional file 1: Table S1a and Table S1b.

The Spearman’s correlation was assessed to determine the relationship among the 18 serum biomarkers and 5 lymphocyte phenotypes of 12 subjects with six visits (total of 71 observations for each marker). There were both positive and negative statistically significant (*p* < 0.05) correlation coefficients (R) among the markers, ranging from very weak to strong as shown in Additional file 2: Table S2. The markers with strong positive rho (r = 0.65, *p* = 0.0001) were serum levels of TNF-α and IL-1ra and markers with strong negative rho (r = − 0.65, *p* = 0.0001) were serum levels of serum sIL-6R and CD4^+^ cells. The markers with weak positive rho (r = 0.24, *p* = 0.043) were serum levels of sCD14 and MIP-1β and the markers with weak negative rho (r = − 0.24, *p* = 0.047) were serum levels of sCD14 and sIL-2R. The correlation for eight markers (rho and *p* values), from strong correlation to no correlation, were depicted in Additional file 3: Figure S1 and Figure S2.

## Discussion

Obtaining a reliable test result requires that all sources of variation should be minimized and components of biological variation (CV_I_ and CV_G_) properly evaluated and managed during the entire process that leads to the final laboratory test report [11].

One of the eventual goals in the study of these biomarkers is to identify those with small biological variation, making them more appropriate for clinical use. It is not surprising that in healthy individuals, biomarker concentrations measured every six months for a period of five years are likely to display greater biological variation than biomarkers measured at a fixed time of the day for a period of 2 to 3 months [12].

The study was done based on the framework of the published study of Ford et al. [13]. Our study has some limitations due to the samples size of twelve participants and short period of follow up of six weeks which were due to practical reasons of financial constraints and keeping all the participants throughout the entire study.

There is little data available regarding biological variability for many of the immunological biomarkers (7 out of 23) examined in this study. Our BV data will provide valuable information for improving the measurement and interpretation of blood biomarker levels examined in our study including setting quality specifications, distinguishing normal levels from those of a diseased state, assessing the usefulness of population-based reference values, selecting the best sample to collect, choosing the best test, validating a new testing procedure, and implementing an internal quality control procedure.

### Biological variation

Available published data for CV_I_ and CV_G_ of immunological biomarkers are presented in Table 5 for comparison purposes [14–17]. Our intra- and inter-individual variation of adiponectin and IL-1β were nearly identical with published data, however there are differences in the intra- and inter-individual variation for other biomarkers compared to ours. Estimates of CV_I_ and CV_G_ should be comparable across all the studies but these differences may be due to the gender ratio, sample size, number of visits, as well as duration of each study.

**Table 5:** Previous studies examining/reporting the intra-individual (CV_I_) and inter-individual (CV_G_) variation of some biomarkers

Biomarker	CV_I_ (%) Pub. Our		CV_G_ (%) Pub. Our		Subject/ Visit/Period (6 m + 6f) / (6v-6w)	Reference
Adiponectin	18.8	15.9	51.2	48.1	(7 m + 15f)/(2v-15mo)	Shand et al. [14]
IL-1β	30.0	30.9	36.0	27.0	(4 m + 11f)/(6v-6mo)	Gonzalez et al. [15]
IL-8	24.0	54.4	31.0	30.2	(4 m + 11f)/(6v-6mo)	Gonzalez et al. [15]
IL-6	48.5	21.5	39.4	26.4	(4 m + 11f)/(6v-6mo)	Cava et al. [16]
TNF-α	43.0	12.7	29.0	27.2	(4 m + 11f)/(6v-6mo)	Gonzalez et al. [15]
sIL-2Rα	5.8	15.1	38.8	41.7	(4 m + 11f)/(6v-6mo)	Cava et al. [16]
sCD163	9.0	15.9	35.9	48.1	(0 m + 12f)/(12v-35d)	Moller et al. [17]

*m* male, *f* female, *v* visit, *w* week, *mo* month, *Pub* published data, *Our* our data, *d* day

Women had higher CV_I_ for 10 out of 18 serum biomarkers compared to men and this could be due to menstrual cycle hormones induced changes. A weakness of the study is that we overlooked the possible influence of menstrual cycle phases’ and this information was not collected from the female participants. There was no significant difference of CV_I_ for blood level of IL-6 reported during menstrual cycle [18] and marked fluctuations of blood levels of TNF-a reported in women [19]. In addition no significant changes reported in lymphocytes subsets over the course of a menstrual cycle [20].

Bear in mind that some of the analytes, notably IFN-γ and IL-1β, are conducted near the minimum detectable dose (MDD) of the assay and several healthy control and HIV-positive subjects of our previously study had plasma levels of markers below MDD. Thus, the low plasma levels of biomarkers may contribute to high CV [21].

### Setting analytical goals based on biological variation

The desirable analytical imprecision goal for monitoring purposes has to be maintained at CV_A_ < 0.50 CV_I_ in order for the amount of variability that is added to true test results to be less than 12% (11.8% calculated). Confidence in the precision of an assay allows us to run fewer internal quality control samples for the biomarker.

The analytical goal of imprecision of biomarkers based on BV are presented in Tables 3 and 4 and by comparing those data with the CV_A_ obtained (Tables 1 and 2) for the biomarkers, we can conclude that, with exception of TNF-α, sCD163, sgp130, sTNF-RII, and CD4^+^ T-cell, all other markers met the desirable analytical precision.

CD4^+^ T-cell, macrophage, and dendritic cells are vulnerable to human immunodeficiency virus type 1 (HIV-1) infection because of their CD4 receptor that the HIV-1 virus uses to infect those cells. The production of infectious virion from provirus requires activation of infected CD4^+^ T-cell and the CD4^+^ T-cell count along with HIV viral load is an important monitoring laboratory tool for follow up of HIV-1 infected patients receiving treatment.

HIV-1 replicates especially in activated CD4^+^ T cell. However, at any given time, most of the CD4^+^ T cells in the HIV-1 infected individual are in resting status and not fully permissible for viral replication. In addition HIV-1 infected individuals receiving highly active antiretroviral therapy (HAART) showing suppression of viremia and activation of CD4^+^ T cells [22]. CD40L that expressed predominantly on activated CD4^+^ T-cells and play multiple role in HIV-1 infection [23]. The blood levels CD4^+^ T-cells and soluble CD40L (sCD40L) could be variable during stages of CD4^+^ T-cell activation by HIV-1 or other infection, but have not seen variability in our normal study population.

The numerical goal for **desirable bias** is also calculated for each of the markers (Tables 3 and 4) and should be less than one quarter of the group biological variation. In a practical sense, the goal for desirable bias is achievable, but it will be problematic when using many different methods for the same assay. These biological variation data of immunological laboratory tests may solve many analytical problems for the assay. In addition, this would provide objective analytical goals for manufacturers of instruments and assay kits [24].

### Reference change values

For a disparity in serial test results to be significant, the difference in numerical results must be greater than the combined variation in the two serial results and this is referred to as the *reference change value (RCV)* [9]*.*

Little intra-individual biological variation for blood immunological biomarkers has been published and laboratories need to determine their own biological variation for intra-individual in order to calculate the RCV. The 95% probability (*p* < 0.05) for immunological biomarkers RCV is presented in Tables 3 and 4 and any differences between two successive quantity results exceeding the RCV for that biomarker should be further investigated [25].

### Utility of reference values

It has well known that CV_I_ is relatively small and mean value of each individual differ from each other and CV_G_ relatively large and also our study illustrated the same pattern for the markers (Figs. 1, 2 and 3). The effect of index of individuality(I.I) of biomarkers on reference values were known and when I. I is less than 0.6, conventional population based reference values are limited in detection of unusual results in contrast when I. I is more than 1.4 [26]. The I. I with exception of IFN-γ (II = 1.52) and IL-8 (II = 1.70) for all other biomarkers in our study were at or below 1.14.

All biomarkers in our study had an index of heterogeneity (IH) of less than 1.58 which is an indication of the homogeneity of the collected specimen for within-subject biological variation. This value determines whether results obtained from a few subjects can be extrapolated to almost all subjects [24, 27].

### Choosing the best test

Knowledge of the biological variation of biomarkers can help us to select the most appropriate test for tracking of disease progression or investigational purposes. In some cases, there may be multiple tests to accomplish the same goal, but one may be superior to the others and BV data can help choose which test to use. For example, serum sCD14, sCD163, and neopterin tests can all be used for the evaluation of macrophage activation and while none of these three tests have diagnostic value, they are useful for monitoring and follow up purposes. Soluble CD14 has a RCV of 23, less than both sCD163 and neopterin, making sCD14 a better monitoring test compared to the other two. However, when considering the ideal test to perform, one must also keep in mind that various stimulants may work on the target cells through different pathways and therefore each biomarker may provide different information about the disease progression.

## Conclusion

The biological variation data generated by our study of 23 biomarkers is the first published data for 16 of the 23 biomarkers and will be valuable for clinical and laboratory use, particularly for setting quality specification goals for immunological biomarker testing purposes. The usefulness of BV data for healthy subjects is well documented for having utility in laboratory medicine. Using serial samples to detect deviations in a patient that differ from those observed in healthy subjects can be a sign of analytic error or real biologic changes. These signals can then be looked into to avoid compromising patient care, or to further investigate the cause of the change [26]. Despite having a limited number of subjects in our study, we found intra-subject variation to be relatively small and inter-subject variation to be relatively large for the majority of biomarkers that were analyzed. In our study, most biomarkers had strong individuality, meaning that the result of a biomarker should be cautiously interpreted if using an established reference values. In general, comparison of a patient’s laboratory results with previous ones will be a better choice.

## Materiel and methods

### Specimens

The study was approved by the institutional review board (IRB) for human studies at UCLA and informed consent was obtained from twelve self-reported healthy volunteers, including six men (21, 32, 33, 40, 42, and 51 years old, with weight of 175, 190, 165, 178, 172, and 194 lbs. and body mass index (BMI) of 25.1, 26.5, 22.4, 27.1, 26.9, and 26.3, respectively) and six women (23, 24, 39, 41, 47, 47 years old, weight of 142, 123, 143, 139, 141, and 140 lbs. and BMI of 24.4, 19.9, 22.4, 23.8, 24.2, and 24.0, respectively). The individuals all self-reported no health problems or menstrual cycle at the time of blood collection. Blood was collected into one 10 mL serum separator tube (SST) and one 4 mL vacutainer tube containing EDTA (Becton Dickinson Vacutainer System Franklin Lakes, NJ) to obtain serum and whole blood, respectively. Blood was collected according to the Clinical and Laboratory Standards Institute (CLSI) standard by phlebotomist between 9:00 am and 11:00 am (to prevent any circadian rhythm variation) once a week from the twelve subjects for a total of six weeks (subject #11 missed visit #4 of his blood draw). The SST blood tube from each individual was centrifuged within one hour after collection (500 *x g* for 10 min) and the serum was separated and frozen at -70 °C until batch analysis. EDTA whole blood was used to determine cell surface markers on lymphocytes by flow cytometry.

### Method of measurement

Serum concentrations of each biomarker were determined using commercially available enzyme- linked immunosorbent assays (ELISA) and all serum samples of each individual were batched and tested in duplicate in a single assay (IL-1β, IL-8, and sIL-6R tested singleton). The detailed instructions of each assay procedure are available through the manufactures’ website and all the assays were performed according to the manufacturer’s instructions. The MDD, intra-assay data of TNF-α, IFN-γ and IL-12p70 and neopterin are from our lab (pool of serum) while the rest of the data is from the manufacturer (R&D Systems) and the method of measurement for each biomarker is summarized in Table 6 while their clinical and research significance along with publication references for each biomarker is included as supplementary text in Additional file 4.

**Table 6:** Suppliers of biomarker assays, minimum detectable dose (MDD), Intra-assay coefficient of variation (CV %), and mean concentration of biomarker sample #1 and 2 of the kits

Supplier of biomarkers		MDD	Intra-Assay Precision		Assayed
*A.*	Cytokines/Chemokines		*Sample #1 (CV%, mean)*	*Sample #2 (CV%, mean)*	
1	Interleukin-1ß (IL-1 ß)^a^	0.033 pg/mL	4.4, 0.315 pg/mL (n = 20)	3.9, 1.30 pg/mL (n = 20)	singleton
1	Interleukin-6 (IL-6)^a^	0.039 pg/mL	6.9, 0.436 pg/mL (n = 20)	7.8, 2.45 pg/mL (n = 20)	duplicate
2	Interleukin-12 (IL-12p70)	2.1 pg/mL	5.0, 15.85 pg/mL (n = 8)	2.13, 45.0 pg/mL (n = 8)	duplicate
1	IL-1 receptor antagonist	6.3 pg/mL	7.3, 66.9 pg/mL (n = 20)	5.0, 607 pg/mL (n = 20)	duplicate
2	Interferon-γ (IFN-γ)	0.03 IU/mL	3.2, 1.26 IU/mL (n = 20)	3.8, 12.28 IU/mL (n = 20)	duplicate
2	Tumor Necrosis Factor-α	3.0 pg/mL	5.2, 86.7 pg/mL (n = 20)	3.7, 591 pg/mL (n = 20)	duplicate
1	Interleukin-8 (IL-8)^a^	0.13 pg/mL	5.5, 5.5 pg/mL (n = 20)	7.3, 37.1 pg/mL(n = 20)	singleton
1	CCL3 (MIP-1 α)	10.0 pg/mL	8.9, 140 pg/mL (n = 20)	8.8, 688 pg/mL (n = 20)	duplicate
1	CCL4 (MIP-1 β)	11.0 pg/mL	9.0, 51.3 pg/mL (n = 20)	3.6, 208 pg/mL (n = 20)	duplicate
1	CCL5 (RANTES)	2.0 pg/mL	2.5, 91.9 pg/mL (n = 20)	1.7, 573 pg/mL (n = 20)	duplicate
B.	Adipocytokines				
1	Adiponectin	0.25 ng/mL	2.5, 19.8 ng/mL (n = 20)	3.4, 69.9 ng/mL (n = 20)	duplicate
1	Leptin	7.8 pg/mL	3.3, 64.5 pg/mL (n = 20)	3.0, 146 pg/mL (n = 20)	duplicate
C.	Soluble Receptors markers				
1	sCD14	125 pg/mL	6.4, 1111 pg/mL (n = 20)	4.8, 2158 pg/mL (n = 20)	duplicate
1	sCD25 (sIL-2Ra)	125 pg/mL	6.1, 207 pg/mL (n = 20)	6.1, 613 pg/mL (n = 20)	duplicate
1	sCD40L	10 pg/mL	5.1, 430 pg/mL (n = 20))	4.5, 1212 pg/mL (n = 20)	duplicate
1	sCD120b (sTNF-RII)	4.2 pg/mL	3.2, 68.7 pg/mL (n = 20)	2.6, 179 pg/mL (n = 20)	duplicate
1	sCD126 (sIL-6R)	0.6 pg/mL	8.6, 134 pg/mL (n = 20)	2.6, 644 pg/mL (n = 20)	singleton
1	sCD130 (spg130)	6.5 pg/mL	4.3, 0.70 ng/mL (n = 20)	5.5, 1.82 ng/mL (n = 20)	duplicate
1	sCD163	0.08 ng/mL	3.8, 20.0 ng/mL (n = 20)	3.4, 35.1 ng/mL (n = 20)	duplicate
D.	Immune Activation markers				
3	Neopterin	2.0 nmol/L	7.4, 4.5 nmol/L (n = 10)	5.6, 18.7 nmol/L (n = 10)	duplicate

^a^High sensitivity ELISA; 1: R&D Systems Inc., USA (A1, B1, C1); 2: Thermo Fisher Scientific, USA (A2); 3: B.R.A.H.M.S., Germany (D3)

### Flow cytometry

Flow cytometric analysis was performed using a FACSCalibur analyzer and CellQuest software (BDIS, San Jose, CA) [28]. In summary for each sample, 50 μl of blood was stained with BD monoclonal antibodies conjugated with immunofluorescence such as fluorescein isothiocyanate (FITC) or phycoerythrin (PE), or Peridinin Chlorophyll Protein Complex (PerCP) or Allophycocyanin (APC), (BD Biosciences, San Jose, CA) using lyse no wash sample preparation method, with lymphocyte gating done according to the manufacturer’s instructions. CD3-FITC, CD8-PE, CD45-PerCP, and CD4-APC were used to label T cells in the first sample. In the second sample, CD3-FITC, CD56/16-PerCP, and CD19-APC monoclonal antibodies were used for identification of natural killer (NK) cells and B cells. The percentages of CD3^+^/CD4^+^ (helper T-cell), CD3^+^/CD8^+^ (cytotoxic T-cell), CD3^−^/CD56/16^+^ (natural killer cell), and CD3^−^/CD19^+^ (B-cell) cells were determined by flow cytometric analysis.

### Statistical analysis

A four-parameter curve-fitting program was used for generation of calibration curves and computation of unknown concentrations of each biomarker. The SigmaPlot software (Jandel Scientific, San Rafael, CA, USA) package was used for all plots. STATA, version 14.23 (StataCorp LLC, College Station, Texas), was used for Spearman’s correlation to assess the relationship among serum levels and cell surface markers. SAS, version 9.3 (SAS Institute, Inc., Cary, North Carolina) was used for ANOVA to calculate intra-individual, inter-individual, and analytical variance (i.e. between duplicates). The data for each marker were checked for outlier results exceeding ±3SD from the mean. One data point of sCD40L (week 5) of a subject was ranked as outlier due to a technical problem and excluded from data analysis. All results of the markers fell within the appropriate range of ±2SD from the mean.

The coefficients of variation (CV) of analytical (CV_A_), intra-individual (CV_I_), and inter-individual (CV_G_) components were calculated according to the approach by Harris and Boyd [29].

The index of individuality (II) is the sample ratio CV_I_ to CV_G_ and was calculated by the following formula:

II = √ (CV_A_^2^ + CV_I_^2^)/CV_G_) for a majority of the biomarkers and simply by CV_I_/CV_G_ for IL-1β, IL-8, sIL-6R, and lymphocyte phenotypes [9]. Examining the heterogeneity of within-subject variation for a given biomarker, the index of heterogeneity (IH) was calculated as the observed CV of the set of individuals’ variance to the theoretical CV using the formula of:

IH = CV_T_ /√(2/[n-1]) where CV_T_ = √ (CV_A_^2^ + CV_I_^2^) and **n** is the average number of observations (samples) per subject. Since the number of samples per subject in our study was 6, an IH < 1.58 indicates that the CV_I_ data is homogeneous for a biomarker because the index is < {1 + 2[1/(2n)^1/2^]} [12, 26]. The critical difference or reference change value **(RCV)** was calculated using the following formula: RCV = 2^1/2^*Z*(CV_A_^2^ + CV_I_^2^)^1/2^ and the Z-score for a probability of 95% or *p* < 0.05 was 1.96 while the Z-score for *p* < 0.01 or probability of 99% was 2.56 (bidirectional).

The reliability coefficient (R) is the ratio of between-subject variation to total variation. It is another measurement of individuality and calculated as: R = CV_G_^2^**/**CV_A_^2^ + CV_I_^2^ + CV_G_^2^.

The value of R ranged between 0 and 1. When **R** approaches 1, this means that there is very little intra-individual variation of results over time, while also indicating that great confidence can be placed in a single result with no need for repeat measurement [30].

The analytical imprecision (CV_A_), bias (B_A_), and total error allowable (TE_a_) for each biomarker based on biological variation for ***desirable*** quality specifications were calculated using the following formulas [11, 31]:

**Precision**: CV_A_<0.50 CV_I_

**Bias**: B_A_<0.250 x (CV_1_² + CV_G_²)^1/2^

**Total Error Allowable** (TE_a_):

For *p*<0.05: TE_a_<0.250(CV_I_^2^+CV_G_^2^)^1/2^+1.65(0.50CV_1_)

For *p*<0.01: TE_a_<0.250(CV_I_^2^+CV_G_^2^)^1/2^+2.33(0.50CV_1_)

For biomarkers in which the *desirable* quality specifications cannot easily be met using general methodology and technology, use of ***minimum*** quality specifications would be appropriate, whereas when they are easily met, ***optimum*** quality specifications should be used [9].

## Additional files

Additional file 1: **Table S1a + b.** The mean, median, and inter quartile range (IQR) values of eighteen serum biomarkers (IL-1β, IL-6, IL-1ra, IFN-γ, TNF-α, IL-8, MIP-1β, RANTES, Adiponectin, Leptin, sCD14, sCD40L, sCD163, spg130, sIL-2Rα, sIL-6R, sTNF-RII, and neopterin) and five lymphocyte phenotypes (CD3, CD4, and CD8 T-cell, CD19 B-cell, and CD56/16 NK cell) were calculated and presented for six males, six females, and both genders. (DOCX 18 kb)

Additional file 2: **Table S2.** The Spearman’s correlation was assessed to determine the relationship among the eighteen serum biomarkers and five lymphocyte phenotypes of 12 subjects with six visits (total of 71 observations for each marker). There were both positive and negative statistically significant (*p* < 0.05) correlation coefficients (r) ranging from very weak to strong as well as instances of no correlation among the biomarkers. (DOCX 18 kb)

Additional file 3: **Figure S1** and **Figure S2.** There were strong positive correlation coefficients (r) between serum levels of IL-1ra and TNF-α (r = 0.65, *p* < 0.001), and percentage CD4+ T-cell and CD3 T-cell (r = 0.49, *p* < 0.001), a strong negative correlation coefficient between serum levels of sIL-6R and percentage of CD4+ T-cell (r = − 0.65, *p* < 0.001), and between percentage of CD56/16 and CD4+ T-cell (r = − 0.58, *p* < 0.001). Moderate correlation was seen between serum levels of MIP-1β and sCD14, between serum levels of sIL-2Rα and sCD14, and between serum levels of IFN-γ and IL-6, while no correlation was seen between serum levels of sCD40 and CD4+ T-cell. (ZIP 2800 kb)

Additional file 4: Method of measurement for each serum biomarker, suppliers of assay kits, and clinical and research significance of each biomarker along with their publication references are presented in Additional file 4. (DOCX 36 kb)

## Data Availability

The data used to support the findings of this study are included within the article and more detailed data of the current study are available from the corresponding author on reasonable request.

## Abbreviations

APC: Allophycocyanin
BMI: Body Mass Index
BV: Biological variation
CD: Cluster of differentiation
CV: Coefficient of variation
CV_A_: Analytical coefficient of variation
CV_G_: Inter-individual coefficient of variation
CV_I_: Intra-individual coefficient of variation
FITC: Fluorescein isothiocyanate
HIV-1: Human immunodeficiency virus type 1
IFN-γ: Interferon gamma
IH: Index of heterogeneity
II: Index of individuality
IL-: Interleukin
IL-1Ra: Interleukin-1 receptor antagonist
MDD: Minimum detectable dose
MIP: Macrophage inflammatory protein
PE: Phycoerythrin
PerCP: Peridinin chlorophyll protein
RCV: Reference change value
RCV: Reference change value
sCD40L: soluble CD40 ligand
sgp130: soluble glycoprotein 130
sIL-2Rα: soluble interleukin-2 receptor alpha
sIL-6R: soluble interleukin-6 receptor
sTNF-RII: soluble tumor necrosis receptor II
TNF-α: tumor necrosis receptor alpha
