# Supplementary material for: Biological variation of immunological blood biomarkers in healthy individuals and quality goals for biomarker tests
Source: BMC Immunol. 2019 Sep 14;20:33. doi: 10.1186/s12865-019-0313-0 (PMC6744707; doi:10.1186/s12865-019-0313-0)
Supplement: Supplementary file 1 — Table S1a + b. The mean, median, and inter quartile range (IQR) values of eighteen serum biomarkers (IL-1β, IL-6, IL-1ra, IFN-γ, TNF-α, IL-8, MIP-1β, RANTES, Adiponectin, Leptin, sCD14, sCD40L, sCD163, spg130, sIL-2Rα, sIL-6R, sTNF-RII, and neopterin) and five lymphocyte phenotypes (CD3, CD4, and CD8 T-cell, CD19 B-cell, and CD56/16 NK cell) were calculated and presented for six males, six females, and both genders. (DOCX 18 kb) [file 12865_2019_313_MOESM1_ESM.docx]

Table S1a. Mean, Median and Inter Quartile Range (IQR) values of immunological biomarkers of eighteen serum biomarkers.

| **Group** | ***Gender*** | ***Male/Female (n=12)*** | | | ***Male(n=6)*** | | | ***Female (n=6)*** | | |
| --- | --- | --- | --- | --- | --- | --- | --- | --- | --- | --- |
|  | ***Biomarker (unit)*** | ***Mean*** | ***Median*** | ***IQR*** | ***Mean*** | ***Median*** | ***IQR*** | ***Mean*** | ***Median*** | ***IQR*** |
| I | IL-1β ( pg/mL) | 0.157 | 0.142 | 0.032 | 0.155 | 0.145 | 0.021 | 0.158 | 0.152 | 0.043 |
|  | IL-6 (pg/mL) | 0.961 | 0.930 | 0.254 | 1.010 | 0.965 | 0.313 | 0.911 | 0.895 | 0.195 |
|  | IL-1Ra (pg/mL) | 419 | 405 | 111 | 330 | 330 | 82 | 507 | 480 | 140 |
|  | IFN-γ ( U/L) | 270 | 265 | 103 | 263 | 258 | 93 | 276 | 273 | 113 |
|  | TNF-α (pg/ml) | 12.63 | 12.43 | 2.18 | 11.99 | 11.92 | 1.95 | 13.27 | 12.93 | 2.41 |
| II | IL-8 (pg/mL) | 12.14 | 11.00 | 3.59 | 10.64 | 10.25 | 3.26 | 13.60 | 11.73 | 3.93 |
|  | MIP-1β (pg/mL) | 101.0 | 98.5 | 20.1 | 99.2 | 97.9 | 7.88 | 103.1 | 99.1 | 15.6 |
|  | RANTES (ng/mL)) | 32.71 | 31.84 | 8.01 | 32.86 | 31.26 | 8.79 | 32.56 | 32.42 | 7.23 |
| III | Adiponectin (μg/mL) | 6.29 | 6.24 | 1.08 | 4.86 | 4.80 | 0.57 | 7.72 | 7.68 | 1.60 |
|  | Leptin (ng/mL) | 7.66 | 7.47 | 1.69 | 4.42 | 4.51 | 0.74 | 10.81 | 10.43 | 2.63 |
| IV | sCD14 (ng/mL) | 1435 | 1428 | 190 | 1459 | 1463 | 212 | 1411 | 1394 | 182 |
|  | sCD40L (ng/mL) | 6.02 | 6.01 | 1.13 | 6.05 | 5.90 | 1.15 | 5.98 | 6.09 | 1.12 |
|  | sCD163 (ng/mL) | 543 | 558 | 66 | 486 | 500 | 58 | 599 | 617 | 74 |
|  | sgp130 (ng/mL) | 281 | 282 | 28 | 309 | 308 | 26 | 253 | 255 | 30 |
|  | sIL-2Rα (pg/mL) | 796 | 793 | 71 | 717 | 736 | 125 | 872 | 850 | 63 |
|  | sIL-6R (ng/mL) | 31.77 | 31.62 | 4.46 | 34.75 | 34.13 | 6.02 | 28.80 | 29.11 | 2.90 |
|  | sTNF-RII (ng/mL) | 2.17 | 2.14 | 0.37 | 2.12 | 2.05 | 0.12 | 2.22 | 2.23 | 0.25 |
| V | Neopterin (nmol/L) | 5.20 | 4.98 | 0.95 | 5.68 | 5.93 | 0.95 | 4.73 | 4.19 | 0.96 |

Table S1b. Mean, Median and Inter Quartile Range (IQR) values of flow cytometry analysis of five lymphocyte phenotypes.

| ***Gender*** | ***Male/Female (n=12)*** | | | ***Male(n=6)*** | | | ***Female (n=6)*** | | |
| --- | --- | --- | --- | --- | --- | --- | --- | --- | --- |
| ***Lymphocyte* (%)** | ***Mean*** | ***Median*** | ***IQR*** | ***Mean*** | ***Median*** | ***IQR*** | ***Mean*** | ***Median*** | ***IQR*** |
| **CD3 (T cells)** | 74 | 74 | 2.75 | 73 | 74 | 2.25 | 74 | 75 | 3.25 |
| **CD4** | 46 | 46 | 3.58 | 40 | 41 | 4.00 | 52 | 52 | 3.83 |
| **CD8** | 25 | 26 | 2.17 | 30 | 31 | 2.67 | 20 | 20 | 1.67 |
| **CD19 (B-cell)** | 12 | 12 | 2.25 | 11 | 11 | 2.50 | 13 | 13 | 2.00 |
| **CD56/16 (NK cell)** | 13 | 13 | 3.58 | 14 | 14 | 3.50 | 12 | 11 | 3.00 |
