# Supplementary material for: Biological variation of immunological blood biomarkers in healthy individuals and quality goals for biomarker tests
Source: BMC Immunol. 2019 Sep 14;20:33. doi: 10.1186/s12865-019-0313-0 (PMC6744707; doi:10.1186/s12865-019-0313-0)
Supplement: Supplementary file 2 — Table S2. The Spearman’s correlation was assessed to determine the relationship among the eighteen serum biomarkers and five lymphocyte phenotypes of 12 subjects with six visits (total of 71 observations for each marker). There were both positive and negative statistically significant (p < 0.05) correlation coefficients (r) ranging from very weak to strong as well as instances of no correlation among the biomarkers. (DOCX 18 kb) [file 12865_2019_313_MOESM2_ESM.docx]

Table S2. Spearman’s correlation coefficient (r_s_) and p values of 71 observations of 23 analytes. Positive and negative correlation presented and gray cells represent no correlation among the markers.

| **Markers** |  |  |  |  |  |  |  |  |  |  |  |  |  |  |  |  |  |  |
| --- | --- | --- | --- | --- | --- | --- | --- | --- | --- | --- | --- | --- | --- | --- | --- | --- | --- | --- |
| **IL-1β** | **IL-1β** |  | | | | | | | | | | | **CD3** | **CD3** |  |  |  |  |
| **IL-1ra** |  | **IL-1ra** |  | | | | | | | | | | **CD4** | **0.4854**  **0.0001** | **CD4** |  |  |  |
| **IL-6** |  |  | **IL-6** |  | | | | | | | | | **CD8** | **0.4156**  **0.0003** | **-0.4816**  **0.0001** | **CD8** |  |  |
| **TNF−α** |  | **0.6475#**  **0.0001** |  | **TNF−α** |  | | | | | | | | **CD19** | **-0.6377**  **0.0001** |  | **-0.4176**  **0.0003** | **CD19** |  |
| **IFN-γ** |  |  | **-0.3353**  **0.0043** |  | **INF−γ** |  | | | | | | | **CD56/16** | **-0.7624**  **0.0001** | **-0.5805**  **0.0001** |  |  | **CD56/16** |
| **Adipo*** |  | **0.4610**  **0.0001** | **0.5077**  **0.0001** |  |  | **Adipo*** |  |  |  |  |  |  |  |  |  |  |  |  |
| **Leptin** |  |  |  |  |  | **0.2465**  **0.0383** | **Leptin** |  |  |  |  |  |  |  |  |  |  |  |
| **IL-8** |  |  | **-0.3888**  **0.0008** | **-0.2824**  **0.0170** |  |  |  | **IL-8** |  |  |  |  |  |  |  |  |  |  |
| **MIP-1β** | **0.4434**  **0.0001** | **0.3715**  **0.0014** |  | **0.2363**  **0.0473** | **-0.2873**  **0.0151** |  |  |  | **MIP-1β** |  |  |  |  |  |  |  |  |  |
| **RANTES** | **-0.2661**  **0.0249** | **0.4933**  **0.0001** | **0.2617**  **0.0275** | **0.5262**  **0.0001** |  | **0.5351**  **0.0001** | **0.2603**  **0.0284** | **-0.3509**  **0.0027** |  | **RANTES** |  |  |  |  |  |  |  |  |
| **sIL-2R** | **0.3048**  **0.0098** |  | **0.2624**  **0.0270** |  |  | **0.4362**  **0.0001** |  |  |  |  | **sIL-2R** |  |  |  |  |  |  |  |
| **sTNF-RII** | **-0.4893**  **0.0001** | **0.4914**  **0.0001** |  | **0.5173**  **0.0001** |  | **0.4246**  **0.0002** | **0.2689**  **0.0233** | **-0.4056**  **0.0004** |  | **0.6044**  **0.0001** |  | **sTNF-RII** |  |  |  |  |  |  |
| **sIL-6R** |  | **-0.3085**  **0.0089** |  |  |  |  |  | **-0.3380**  **0.0039** |  |  |  |  | **sIL-6R** |  |  |  |  |  |
| **spg130** | **0.2949**  **0.0125** | **-0.3268**  **0.0054** |  | **-0.2546**  **0.0321** |  |  | **-0.4430**  **0.0001** |  |  | **-0.4027**  **0.0005** | **0.4011**  **0.0005** | **-0.4915**  **0.0001** | **0.4308**  **0.0002** | **spg130** |  |  |  |  |
| **NPT** | **0.3157**  **0.0073** |  |  | **0.2889**  **0.0146** |  |  | **0.3603**  **0.0020** |  | **0.5504**  **0.0001** |  |  |  |  |  | **NPT** |  |  |  |
| **sCD14** |  |  |  |  | **-0.3825**  **0.0010** |  |  |  | **0.2351**  **0.0484** |  | **-0.2367**  **0.0469** |  |  | **-0.2380**  **0.0456** | **0.4239**  **0.0002** | **sCD14** |  |  |
| **sCD40L** |  |  | **-0.2563**  **0.0322** |  |  | **-0.3921**  **0.0008** |  |  |  |  | **-0.5270**  **0.0001** |  |  | **-0.4305**  **0.0002** |  |  | **sCD40L** |  |
| **sCD163** |  | **0.4666**  **0.0001** |  | **0.6134**  **0.0001** |  | **0.3061**  **0.0094** | **0.6525**  **0.0001** |  | **0.3474**  **0.0030** | **0.3946**  **0.0007** |  | **0.3006**  **0.0109** |  |  | **0.5486**  **0.0001** | **0.2451**  **0.0394** |  | **sCD163** |
| **CD3** |  |  |  | **-0.3914**  **0.0007** |  | **0.4355**  **0.0001** |  |  |  |  |  |  | **-0.3014**  **0.0106** |  |  | **0.2656**  **0.0252** | **-0.2844**  **0.0170** |  |
| **CD4** |  | **0.3157**  **0.0073** |  |  |  | **0.5223**  **0.0001** |  |  |  | **0.2426**  **0.0415** |  |  | **-0.6532**  **0.0001** | **-0.4464**  **0.0001** |  |  |  |  |
| **CD8** |  | **-0.4062**  **0.0004** |  | **-0.3371**  **0.0040** |  |  |  |  |  | **-0.3017**  **0.0106** | **-0.2690**  **0.0233** |  | **0.4032**  **0.0005** | **0.3960**  **0.0006** |  |  |  |  |
| **CD19** |  |  |  |  | **0.3139**  **0.0077** | **-0.3872**  **0.0008** | **-0.2610**  **0.0279** |  |  |  |  |  |  |  | **-0.3134**  **0.0078** | **-0.2512**  **0.0346** |  | **-0.3282**  **0.0052** |
| **CD56/16** |  |  |  | **0.5525**  **0.0001** |  |  |  | **-0.3266**  **0.0054** |  | **0.2573**  **0.0303** |  |  | **0.5190**  **0.0001** |  |  | **-0.2481**  **0.0370** |  | **0.2436**  **0.0406** |

*: Adiponectin, #: top number is correlation (r) and bottom number is significance or p value.
