# Supplementary figures and images for: Biological variation of immunological blood biomarkers in healthy individuals and quality goals for biomarker tests
Source: BMC Immunol. 2019 Sep 14;20:33. doi: 10.1186/s12865-019-0313-0 (PMC6744707; doi:10.1186/s12865-019-0313-0)

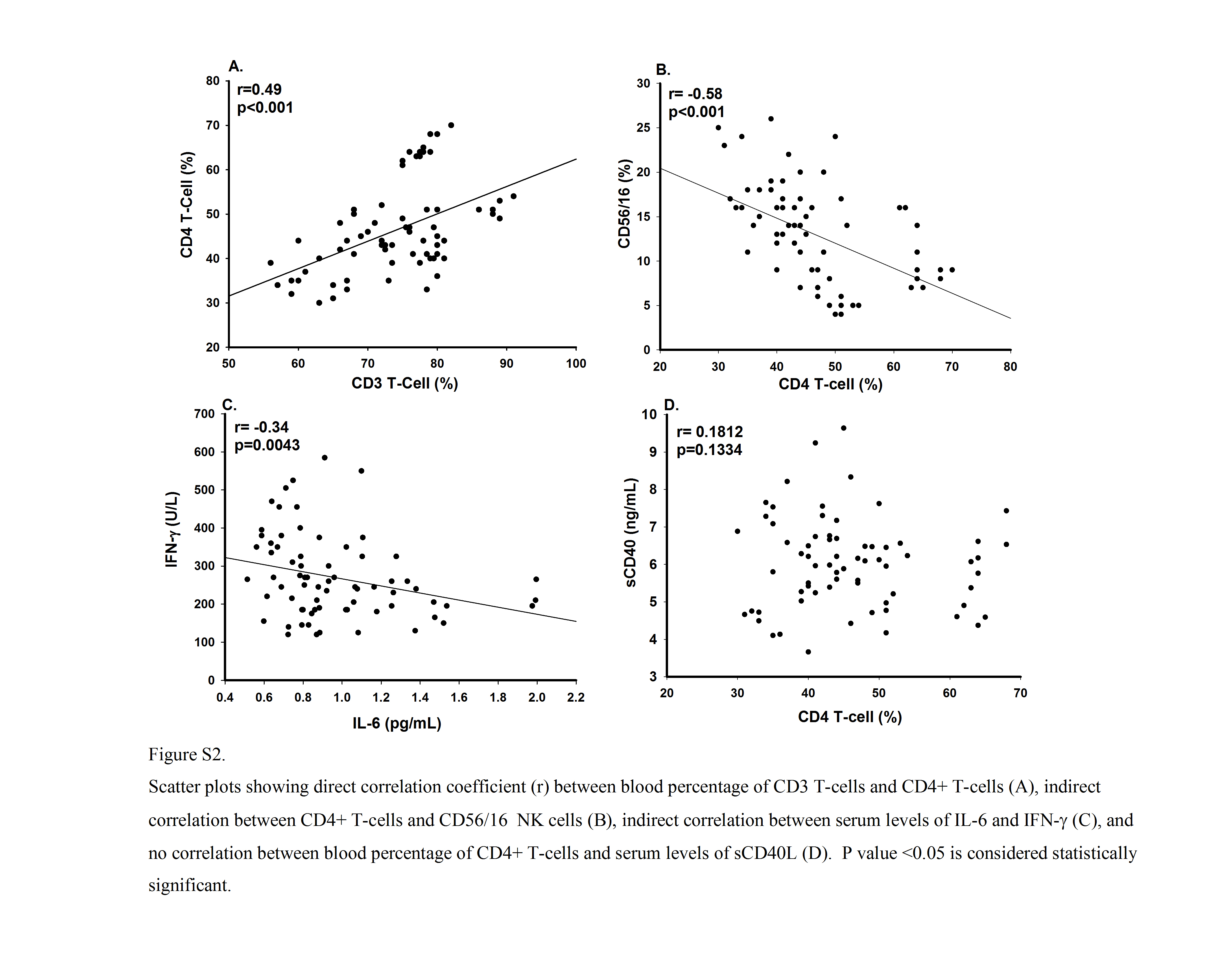

Supplement: Supplementary file 3 — Figure S1 and Figure S2. There were strong positive correlation coefficients (r) between serum levels of IL-1ra and TNF-α (r = 0.65, p < 0.001), and percentage CD4+ T-cell and CD3 T-cell (r = 0.49, p < 0.001), a strong negative correlation coefficient between serum levels of sIL-6R and percentage of CD4+ T-cell (r = − 0.65, p < 0.001), and between percentage of CD56/16 and CD4+ T-cell (r = − 0.58, p < 0.001). Moderate correlation was seen between serum levels of MIP-1β and sCD14, between serum levels of sIL-2Rα and sCD14, and between serum levels of IFN-γ and IL-6, while no correlation was seen between serum levels of sCD40 and CD4+ T-cell. (ZIP 2800 kb) [file 12865_2019_313_MOESM3_ESM.zip › Fig S2.JPG]

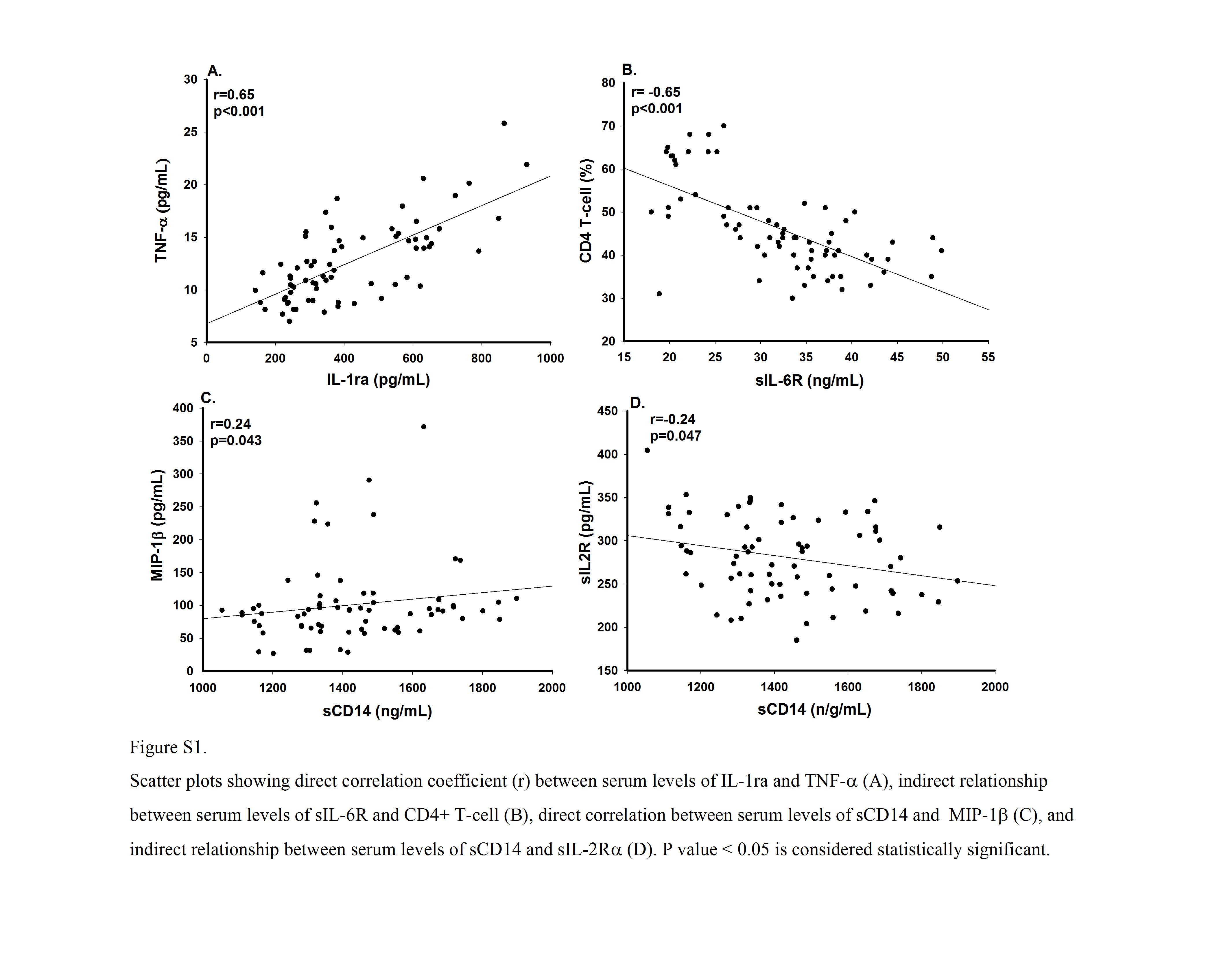

Supplement: Supplementary file 3 — Figure S1 and Figure S2. There were strong positive correlation coefficients (r) between serum levels of IL-1ra and TNF-α (r = 0.65, p < 0.001), and percentage CD4+ T-cell and CD3 T-cell (r = 0.49, p < 0.001), a strong negative correlation coefficient between serum levels of sIL-6R and percentage of CD4+ T-cell (r = − 0.65, p < 0.001), and between percentage of CD56/16 and CD4+ T-cell (r = − 0.58, p < 0.001). Moderate correlation was seen between serum levels of MIP-1β and sCD14, between serum levels of sIL-2Rα and sCD14, and between serum levels of IFN-γ and IL-6, while no correlation was seen between serum levels of sCD40 and CD4+ T-cell. (ZIP 2800 kb) [file 12865_2019_313_MOESM3_ESM.zip › Fig. S1.JPG]
