# Supplementary material for: Biological variation of immunological blood biomarkers in healthy individuals and quality goals for biomarker tests
Source: BMC Immunol. 2019 Sep 14;20:33. doi: 10.1186/s12865-019-0313-0 (PMC6744707; doi:10.1186/s12865-019-0313-0)
Supplement: Supplementary file 4 — Method of measurement for each serum biomarker, suppliers of assay kits, and clinical and research significance of each biomarker along with their publication references are presented in Additional file 4. (DOCX 36 kb) [file 12865_2019_313_MOESM4_ESM.docx]

**Additional File 4: Biomarkers methods data, suppliers and clinical and research significant**

**I. Cytokine**

- **IL-1β** concentrations were measured using a high sensitivity sandwich enzyme immunoassay from R&D Systems (Minneapolis, MN). The mean minimum detectable dose (MDD) was 0.033 pg/mL and the intra-assay coefficient of variation (CV) was determined to be 4.4% and 2.4% for assay control samples at mean concentrations of 0.315 pg/mL (n=20) and 2.52 pg/mL (n=20), respectively.

**Clinical and research significance**: It is a pro-inflammatory cytokine, plays a role in normal homeostasis, and has also been associated with bone formation, insulin secretion, appetite regulation, fever reduction, and neuronal development. The inflammatory response is deemed to be responsible for development of major chronic diseases that are highly prevalent in the elderly [1].

- **IL-6** concentrations were measured using a high sensitivity sandwich enzyme immunoassay from R&D Systems. The mean MDD was 0.039 pg/mL and the intra-assay CV was determined to be 6.9% and 7.4% for assay control samples at mean concentrations of 0.436 pg/mL (n=20) and 5.53 pg/mL (n=20), respectively.

**Clinical and research significance**: It is a pro-inflammatory cytokine and stimulates the inflammatory and auto-immune processes in many diseases such as [diabetes](https://en.wikipedia.org/wiki/Diabetes) [2], [atherosclerosis](https://en.wikipedia.org/wiki/Atherosclerosis) [3], [depression](https://en.wikipedia.org/wiki/Major_depressive_disorder) [4], [Alzheimer's Disease](https://en.wikipedia.org/wiki/Alzheimer%27s_Disease) [5], [systemic lupus erythematosus](https://en.wikipedia.org/wiki/Systemic_lupus_erythematosus) [6], [multiple myeloma](https://en.wikipedia.org/wiki/Multiple_myeloma) [7], [prostate cancer](https://en.wikipedia.org/wiki/Prostate_cancer) [8], [Bechet’s disease](https://en.wikipedia.org/wiki/Beh%C3%A7et%27s_disease) [9], and [rheumatoid arthritis](https://en.wikipedia.org/wiki/Rheumatoid_arthritis) [10].

- **IL-12p70** concentrations were measured using a sandwich enzyme immunoassay from Bender MedSystems GmbH (Vienna Austria) and distributed by Thermo Fisher Scientific USA. The mean MDD was 2.1 pg/mL and the intra-assay CV was determined to be 5.0% and 2.1% for assay control samples at mean concentrations of 15.9 pg/mL (n=6) and 45.0 pg/mL (n=6), respectively.

**Clinical and research significance:** IL-12 p70 is a pro-inflammatory cytokine that induces production of IFN-γ which favors the differentiation of Type I helper T-cells and forms a link between innate resistance and adaptive immunity [11]. Increased levels of IL-12 are seen in type 2 diabetes patients with higher insulin resistance [12] and Graves’ disease and levels decrease during treatment [13].

- **IL-1Ra** concentrations were measured using a sandwich enzyme immunoassay technique from R&D Systems. The mean MDD was 6.3 pg/mL and the intra-assay CV was determined to be 7.3% and 5.0% for assay control samples with mean concentrations of 66.9 pg/mL (n=20) and 607.0 pg/mL (n=20), respectively.

**Clinical and research significance:** It is a member of the interleukin-1 cytokine family and secreted by various types of cells including immune cells, epithelial cells, and adipocytes. It is a natural inhibitor of the pro-inflammatory effect of IL-1. IL-1Ra is used in the treatment of rheumatoid arthritis, an autoimmune disease in which IL-1 plays a key role, and a human recombinant form of IL-1Ra (anakinra) is commercially available for treatment. IL-1Ra gene polymorphism is reported to be associated with increased risk of osteoporotic fractures [14] and gastric cancer [15].

- **IFN-γ** concentrations were measured using a solid phase enzyme amplified sensitivity immunoassay from BioSource Europe SA (Nivelles, Belgium) and distributed by Thermo Fisher Scientific USA. The assay utilizes an oligoclonal system in which several monoclonal antibodies are directed against distinct epitopes of IFN-γ. The mean MDD was 0.03 IU/mL and the intra-assay CV was determined to be 3.2% and 3.8% for assay control samples at mean concentrations of 1.26 IU/mL (n=20) and 12.28 IU/mL (n=20), respectively.

**Clinical and research significance:** **IFN-γ** is an important immunoregulatory cytokine which plays key roles in the host defense through anti-viral, anti-proliferative, and immunoregulatory activities [16]. It induces production of cytokines and up-regulates the expression of various membrane proteins including class I and class II MHC antigen, FC receptor, leukocyte adhesion molecules, and B7 family antigen. Also IFN-γ is a potent activator of macrophage effector functions which results in increased plasma levels of neopterin [17].

- **TNF-α** concentrations were measured using a solid phase enzyme amplified sensitivity immunoassay from BioSource Europe SA (Nivelles, Belgium) and distributed by Thermo Fisher Scientific USA. The assay utilizes an oligoclonal system in which several monoclonal antibodies are directed against distinct epitopes of TNF-α. The mean MDD of the assay was 3.0 pg/mL and the intra-assay CV was determined to be 8.1% and 5.5% for assay control samples with mean concentrations of 8.39 pg/mL (n=10) and 37.8 pg/mL (n=10), respectively.

**Clinical and research significance:** TNF-α is a cell signaling cytokine involved in systemic inflammation. High blood levels of this cytokine are seen in a wide variety of pathological conditions including asthma [18], Crohn’s disease [19], rheumatoid arthritis [20], neuropathic pain [21], obesity [22], type 2 diabetes [23], septic shock [24], autoimmunity [25], and cancer [26].

**II.** **Chemokine**

- **IL-8 also known as CXCL8** concentrations were measured using a high sensitivity sandwich enzyme immunoassay from R&D Systems. The mean MDD was 0.13 pg/mL and the intra-assay CV was determined to be 5.5% and 7.3% for assay control samples with mean concentrations of 5.5 pg/mL (n=20) and 37.1 pg/mL (n=20), respectively.

**Clinical and research significance:** A variety of cells secrete IL-8 including monocytes and neutrophils [27]. It has a pro-inflammatory effect and is involved in angiogenesis and the pathogenesis of atherosclerosis and cancers [28-29].

- **MIP-1α also known as CCL3** concentrations were measured using a sandwich enzyme immunoassay from R&D Systems. The MDD was 10.0 pg/mL and the intra-assay CV was determined to be 8.9% and 8.8% for assay control samples with mean concentrations of 140.0 pg/mL (n=20) and 688.0 pg/mL (n=20), respectively.

**Clinical and research significance: see CCL5**

- **MIP-1β** **also known as CCL4** concentrations were measured using a sandwich enzyme immunoassay from R&D Systems. The MDD was 11.0 pg/mL and the intra-assay CV was determined to be 9.0 % and 3.2% for assay control samples with mean concentrations of 51.30 pg/mL (n=20) and 932.0 pg/mL (n=20), respectively.

**Clinical and research significance: see CCL5**.

- **RANTES also known as CCL5** concentrations were measured using a sandwich enzyme immunoassay from R&D Systems. The mean MDD was 2.0 pg/mL and the intra-assay CV was determined to be 3.6% and 2.4% for assay control samples with mean concentrations of 108 pg/mL (n=20) and 599 pg/mL (n=20), respectively.

**Clinical and research significance:** Chemokines are secondary pro-inflammatory mediators that are induced by primary pro-inflammatory cytokines and have been shown to possess chemoattractant activity and play key roles in immunoregulatory and inflammatory processes. They are major regulators of leukocyte traffic (adhesion, chemotaxis, and activation) during normal and inflammation conditions. Their action is mediated by the receptors on (target cells) monocytes/macrophages, T-cells, NK cells, basophils, immature dendritic cells, and bone marrow cells [30]. MIP-1α, MIP-1β, and RANTES compete with HIV-1 for the CCR5 receptor on T-cells. All three chemokines have been implicated as possible HIV suppressive factors produced by CD8+ T-cells [31], they also play a role in cirrhotic patients and liver cancer [32].

**III. Adipocytokines**

- **Adiponectin** concentrations were measured using a sandwich enzyme immunoassay from R&D Systems (Minneapolis, MN). The mean MDD was 0.246 ng/mL and the intra-assay CV was determined to be 2.5% and 4.7% for assay control samples with mean concentrations of 19.8 ng/mL (n=20) and 143.0 ng/mL (n=20), respectively.

**Clinical and research significance: Adiponectin** may have a role in the development of various diseases of the circulatory system and high plasma levels of adiponectin may be linked to increased mortality in patients with heart failure. Conversely, adiponectin may have a suppressive effect on inflammatory cytokines, potentially making it beneficial in treatment and prevention of atherosclerosis [33].

- **Leptin** concentrations were measured using a sandwich enzyme immunoassay from R&D Systems. The MDD was 7.8 pg/mL and the intra-assay CV was determined to be 3.3% and 3.2% for assay control samples with mean concentrations of 64.5 pg/mL (n=20) and 621.0 pg/mL (n=20), respectively.

**Clinical and research significance:** It is an adipocyte-derived hormone that is essential for normal body weight regulation. Serum levels of leptin are elevated in cases of cardiovascular disease, hypertension, congestive heart failure, and myocardial infraction. Blood levels of leptin also significantly correlate with (Homeostatic Model Assessment for Insulin Resistance) HOMA-IR which reflects the degrees of insulin resistance with the concentration of blood insulin [34].

**IV. Soluble receptors markers**

- **sCD14** concentrations were measured using a sandwich enzyme immunoassay from R&D Systems. The mean MDD was 125.0 pg/mL and the intra-assay CV was determined to be 6.4% and 5.2% for assay control samples with mean concentrations of 1111 ng /mL (n=20) and 4187 ng/mL (n=20), respectively.

**Clinical and research significance:** sCD14 is secreted by the liver, monocytes, macrophages, and to a lesser extent by neutrophils. It is also present in human milk, where it is believed to regulate microbial growth in the infant gut. Serum level measurements of sCD14 are useful for prediction of pulmonary exacerbation in cystic fibrosis [35] and independently predict mortality and accelerated disease progression in HIV-1 infection [36].

- **sCD25 also known as** **IL-2 Rα** concentrations were measured using a sandwich enzyme immunoassay from R&D Systems. The MDD was 10.0 pg/mL and the intra-assay CV was determined to be 6.1% and 4.6% for assay control samples with mean concentrations of 207 pg/mL (n=20) and 2357 pg/mL (n=20), respectively. For converting sample values obtained with the sIL-2 Rα kit to approximate NIBSC 97/600 units, use the equation of approximate value (IU/mL) = 0.0602 x sIL-2 Rα value (pg/mL).

**Clinical and research significance:** IL-2R is expressed on all T-cells and released into the surrounding extracellular fluid as soluble IL-2R after activation of cells due to infection [37], cancers [38-39], and transplantation [40] and autoimmune disease [41].

- **sCD40L** concentrations were measured using a sandwich enzyme immunoassay from R&D Systems. The mean MDD 4.2 pg/mL and the intra-assay CV was determined to be 5.1% and 5.4% for assay control samples with mean concentrations of 430 pg/mL (n=20) and 2638 pg/mL (n=20), respectively.

**Clinical and research significance:** CD40L is expressed predominantly on activated CD4+ T-cells and other cells such as NK cells, mast cells, basophils, and eosinophils. Elevated levels of sCD40L have been observed in the sera of patients with systemic lupus erythematosus [42], chronic lymphocytic leukemia [43], and unstable angina [44].

- **sCD120b** **also known as** **sTNF-RII** concentrations were measured using a sandwich enzyme immunoassay from R&D Systems. The mean MDD was 0.6 pg/mL and the intra-assay CV was determined to be 3.2% and 4.8% for assay control samples with mean concentrations of 68.7 pg/mL (n=20) and 349.0 pg/mL (n=20), respectively.

**Clinical and research significance:** Elevated blood levels of sTNF-RII have been seen in HIV infection [45], endotoxinemia [46], meningiococcemia [47], rheumatoid arthritis [48-49], inflammatory bowel disease [50], spinal instrumentation surgery [51], and traffic air pollution [52].

- **sCD126 also known as IL-6R** concentrations were measured using a sandwich enzyme immunoassay from R&D Systems. The mean MDD was 6.5 pg/mL and the intra-assay CV was determined to be 8.6% and 2.3% for assay control samples with mean concentrations of 134 pg/mL (n=20) and 1669 pg/mL (n=20), respectively.

**Clinical and research significance: IL-6R** is a type I cytokine receptor and is expressed on the surface of monocytes, neutrophils, and B cells. It is present in the plasma of healthy individuals and elevated levels of this soluble receptor have been detected in numerous disease states. Thus, sIL-6R has the potential to regulate both local and systemic by forming a ligand-receptor complex with IL-6 to stimulate proliferation, differentiation and inflammatory process [53].

- **sgp130** concentrations were measured using a sandwich enzyme immunoassay from R&D Systems. The MDD was 0.05 ng/mL and the intra-assay CV was determined to be 4.3% and 4.7% for assay control samples with mean concentrations of 0.70 ng/mL (n=20) and 8.43 ng/mL (n=20), respectively.

**Clinical and research significance:** IL-6 exerts its activity through binding to a high affinity receptor complex which consists of two membrane glycoproteins: IL-6R and sgp130. This regulatory complex system is involved in the modulation of the biological activities of IL-6 under normal and pathological conditions. Soluble gp130 plays a critical role in prostate cancer invasion and higher plasma levels of sgp130 are associated with the features of biologically aggressive prostate cancer as well as progression [54].

- **sCD163** concentrations were measured using a sandwich enzyme immunoassay from R&D Systems. The mean MDD was 0.117 ng/mL and the intra-assay CV was determined to be 3.8% and 3.5% for assay control samples with mean concentrations of 20 ng/mL (n=20) and 65.6 ng/mL (n=20), respectively.

**Clinical and research significance:** sCD163 is a monocyte/macrophage activation marker and it is essential for clearance of hemoglobin-haptoglobin (Hb-Hp) complexes in the liver, spleen, and in circulation [55]. sCD163 also has a protective and anti-inflammatory role as shown by its ability to inhibit phorbol ester-induced lymphocyte proliferation [56]. Decreased production of cellular CD163 within atherosclerotic plaques and increased circulating sCD163 may occur in diabetes mellitus [57]. Elevated levels of sCD163 has been reported in multiple sclerosis [58] and is also associated with non-calcified coronary plaques in men with chronic HIV infection and low or undetectable viremia [59]. High levels of sCD163 predict mortality in acute liver failure [60].

**V. Immune activation marker**

- **Neopterin (NPT)** concentrations were measured using a competitive enzyme immunoassay from BRAHMS (Berlin, Germany). The lower limit of detection was 1 nmol/L and the intra-assay CV was 4.1% and 6.6% for assay control samples with mean concentrations of 7.93 nmol/L (n=12) and 23.8 nmol/L (n=12), respectively.

**Clinical and research significance:** Increased amounts of neopterin are produced by monocyte and macrophages upon stimulation by IFN-γ**.** Measurement of serum levels of neopterin is useful for monitoring cell-mediated immunity and blood levels of neopterin are increased prior to infection by viruses such as HIV-1 [61-62].

**Reference**

1. Di Iorio A, Ferrucci L, Sparvieri E, et al. Serum IL-1 beta Levels in health and disease: a population-based study. The In CHIANTI study. Cytokine. 2003; 21;22(6):198-2
2. Kristiansen OP, Mandrup-Poulsen T. "Interleukin-6 and diabetes: the good, the bad, or the indifferent?". Diabetes. 2005; 54 Suppl 2: S11424.
3. Dubiński A, Zdrojewicz Z "The role of interleukin-6 in development and progression of atherosclerosis". Pol. Merkur. Lekarski (in Polish) 2007; 22 (130): 291–4.
4. owlati Y, Herrmann N, Swardfager W, et al. "A meta-analysis of cytokines in major depression". Biol. Psychiatry 2010; 67 (5): 446–57.
5. Swardfager W, Lanctôt K, Rothenburg L, et al. "A meta-analysis of cytokines in Alzheimer's disease". Biol. Psychiatry 2010; 68 (10): 930–41.
6. Tackey E, Lipsky PE, Illei "Rationale for interleukin-6 blockade in systemic lupus erythematosus". Lupus 2004; 13 (5): 339–43.
7. Gadó K, Domján G, Hegyesi H, Falus A "Role of INTERLEUKIN-6 in the pathogenesis of multiple myeloma". Cell Biol. Int.2000; 24 (4): 195–209.
8. Smith PC, Hobisch A, Lin DL, et al. "Interleukin-6 and prostate cancer progression". Cytokine Growth Factor Rev. 2001; 12 (1): 33–40.
9. Hirohata S, Kikuchi H “Changes in biomarkers focused on differences in disease course or treatment in patients with neuro-Behçet's disease".Intern. Med.2012; 51 (24): 3359–65.
10. Nishimoto N. "Interleukin-6 in rheumatoid arthritis". Curr Opin Rheumatol 2006; 18 (3): 277–81.
11. Trinchieri G. Interleukin-12 and the regulation of innate resistance and adaptive immunity. Nat Rev Immunol. 2003; 3(2):133-46
12. Mishra M, Kumar H, Bajpai S, et al. Level of serum IL-12 and its correlation with endothelial dysfunction, insulin resistance, proinflammatory cytokines and lipid profile in newly diagnosed type 2 diabetes. Diabetes Res Clin Pract. 2011; 94(2):255-61
13. Tamaru M, Matsuura B, Onji M. Increased levels of serum interleukin-12 in Graves' disease. Eur J Endocrinol. 1999; 141(2):111-6.
14. Langdahl BL, Løkke E, Carstens M, et al . "Osteoporotic fractures are associated with an 86-base pair repeat polymorphism in the interleukin-1 receptor antagonist gene but not with polymorphisms in the interleukin-1beta gene". J. Bone Miner. Res.2000; 15 (3): 402–14.
15. El-Omar EM, Carrington M, Chow WH, et al. "Interleukin-1 polymorphisms associated with increased risk of gastric cancer". Nature 2010; 404 (6776): 398–402
16. Schoenborn JR, Wilson CB. Regulation of interferon-gamma during innate and adaptive immune responses. Adv Immunol. 2007; 96:41-101. Review.
17. Huber C, Batchelor JR, Fuchs D, Hausen A, Lang A, Niederwieser D, Reibnegger G, Swetly P, Troppmair J, Wachter H., Immune response-associated production of neopterin. Release from macrophages primarily under control of interferon-gamma. J Exp Med. 1984; 160(1):310-6.
18. Berry M, Brightling C, Pavord I, Wardlaw A. TNF-alpha in asthma. Curr Opin Pharmacol. 2007; 7(3):279-82. Epub 2007 May 1. Review
19. D'Haens G. Anti-TNF therapy for Crohn's disease. Curr Pharm Des. 2003; (4):289-94.
20. Feldmann M, Maini RN. Anti-TNF alpha therapy of rheumatoid arthritis: what have we learned? Annu Rev Immunol. 2001; 19:163-96. Review
21. Leung L, Cahill CM. TNF-alpha and neuropathic pain--a review. J. Neuroinflammation. 2010; 7:27. Review
22. Tzanavari T, Giannogonas P, Karalis KP. TNF-alpha and obesity. Curr Dir Autoimmun. 2010; 11:145-56. Review.
23. Swaroop JJ, Rajarajeswari D, Naidu JN. Association of TNF-α with insulin resistance in type 2 diabetes mellitus. Indian J Med Res. 2012; 135:127-30.
24. Riché FC, Cholley BP, Panis YH, et al. Inflammatory cytokine response in patients with septic shock secondary to generalized peritonitis. Crit Care Med. 2000; 28(2):433-7
25. Loftus EV Jr. Biologic therapy in Crohn's disease: review of the evidence. Rev Gastroenterol Disord. 2007; 7 Suppl 1:S3-12. Review
26. Wajant H. The role of TNF in cancer. Results Probl Cell Differ. 2009; 49:1-15.
27. Smedman C, Gårdlund B, Nihlmark K, Gille-Johnson P, Andersson J, Paulie S.

ELISpot analysis of LPS-stimulated leukocytes: human granulocytes selectively secrete IL-8, MIP-1beta and TNF-alpha. J Immunol Methods. 2009 ; 346(1-2):1-8

1. Apostolakis S, Vogiatzi K, Amanatidou V, Spandidos DA. Interleukin 8 and cardiovascular disease. Cardiovasc Res. 2009; 84(3):353-60.
2. Singh S, Singh AP, Sharma B, Owen LB, Singh RK. CXCL8 and its cognate receptors in melanoma progression and metastasis. Future Oncol. 2010
3. Raport CJ, Gosling J, Schweickart VL, Gray PW, Charo IF. Molecular cloning and functional characterization of a novel human CC chemokine receptor (CCR5) for RANTES, MIP-1beta, and MIP-1alpha. J Biol Chem. 1996; 271(29):17161-6.
4. Cocchi F, DeVico AL, Garzino-Demo A, Arya SK, Gallo RC, Lusso P., Identification of RANTES, MIP-1 alpha, and MIP-1 beta as the major HIV-suppressive factors produced by CD8+ T cells. Science. 1995; 270(5243):1811-5
5. Sadeghi M, Lahdou I, Oweira H, Daniel V, Terness P, Schmidt J, Weiss KH, Longerich T, Schemmer P, Opelz G, Mehrabi A. Serum levels of chemokines CCL4 and CCL5 in cirrhotic patients indicate the presence of hepatocellular carcinoma. Br J Cancer. 2015; 113(5):756-62.
6. Shibata R, Ouchi N, Murohara T, Adiponectin and cardiovascular Disease, Circulation Journal 2009; 73:608-614.
7. Esteghamati A, Khalilzadeh O, Anvari M, Rashidi A, Mokhtari M, Nakhjavani M., Association of serum leptin levels with homeostasis model assessment-estimated insulin resistance and metabolic syndrome: the key role of central obesity. Metab Syndr Relat Disord. 2009; 7(5):447-52
8. Quon BS, Ngan DA, Wilcox PG, Man SF, Sin DD. Plasma sCD14 as a biomarker to predict pulmonary exacerbations in cystic fibrosis. PLoS One. 2014; 9(2):e8934113
9. Sandler NG, Wand H, Roque A, Law M, Nason MC, Nixon DE, Pedersen C, Ruxrungtham K, Lewin SR, Emery S, Neaton JD, Brenchley JM, Deeks SG, Sereti I, Douek DC; INSIGHT SMART Study Group. Plasma levels of soluble CD14 independently predict mortality in HIV infection. J Infect Dis. 2011 Mar 15;203(6):780-90.
10. Bass HZ, Nishanian P, Hardy WD, Mitsuyasu RT, Esmail E, Cumberland W, Fahey JL. Immune changes in HIV-1 infection: significant correlations and differences in serum markers and lymphoid phenotypic antigens. Clin Immunol Immunopathol. 1992; 64(1):63-70
11. Masuda A, Arai K, Nishihara D, Mizuno T, Yuki H, Kambara T, Betsunoh H, Abe H, Yashi M, Fukabori Y, Yoshida K, Kamai T. Clinical significance of serum soluble T cell regulatory molecules in clear cell renal cell carcinoma. Biomed Res Int. 2014; 2014:396064.
12. Yoshida N, Oda M, Kuroda Y, Katayama Y, Okikawa Y, Masunari T, Fujiwara M, Nishisaka T, Sasaki N, Sadahira Y, Mihara K, Asaoku H, Matsui H, Seto M, Kimura A, Arihiro K, Sakai A. Clinical significance of sIL-2R levels in B-cell lymphomas. PLoS One. 2013; 8(11) e78730
13. Döring M, Cabanillas Stanchi KM, Mezger M, Erbacher A, Feucht J, Pfeiffer M,Lang P, Handgretinger R, Müller I. Cytokine serum levels during post-transplant adverse events in 61 pediatric patients after hematopoietic stem cell transplantation. BMC Cancer. 2015; 15:607
14. Barak V, Selmi C, Schlesinger M, Blank M, Agmon-Levin N, Kalickman I, Gershwin ME, Shoenfeld Y. Serum inflammatory cytokines, complement components, and soluble interleukin 2 receptor in primary biliary cirrhosis. J Autoimmun. 2009; 33(3-4):178-82
15. Vakkalanka RK, Woo C, Kirou KA, Koshy M, Berger D, Crow MK. Elevated levels and functional capacity of soluble CD40 ligand in systemic lupus erythematosus sera. Arthritis Rheum. 1999; 42(5):871-81
16. Younes A, Snell V, Consoli U, Clodi K, Zhao S, Palmer JL, Thomas EK, Armitage RJ, Andreeff M. Elevated levels of biologically active soluble CD40 ligand in the serum of patients with chronic lymphocytic leukaemia. Br J Haematol. 1998; 100(1):135-41
17. Aukrust P, Müller F, Ueland T, Berget T, Aaser E, Brunsvig A, Solum NO, Forfang K, Frøland SS, Gullestad L. Enhanced levels of soluble and membrane-bound CD40 ligand in patients with unstable angina. Possible reflection of T lymphocyte and platelet involvement in the pathogenesis of acute coronary syndromes. Circulation. 1999; 100 (6):614-20.
18. Aziz N, Nishanian P, Fahey JL. Levels of cytokines and immune activation markers in plasma in human immunodeficiency virus infection: quality control procedures. Clin Diagn Lab Immunol. 1998; 5(6):755-61.
19. Spinas GA, Keller U, Brockhaus M. Release of soluble receptors for tumor necrosis factor (TNF) in relation to circulating TNF during experimental endotoxinemia. J Clin Invest. 1992; 90(2):533-6.
20. Girardin E, Roux-Lombard P, Grau GE, Suter P, Gallati H, Dayer JM. Imbalance between tumour necrosis factor-alpha and soluble TNF receptor concentrations in severe meningococcaemia. The J5 Study Group. Immunology. 1992; 76(1):20-3.
21. Lopatnikova JA, Golikova EA, Shkaruba NS, Sizikov AE, Sennikov SV. Analysis of the levels of tumor necrosis factor (TNF), autoantibodies to TNF, and soluble TNF receptors in patients with rheumatoid arthritis, Scand J Rheumatol. 2013; 42(6): 429-32.
22. Tolusso B, Sacco S, Gremese E, La Torre G, Tomietto P, Ferraccioli GF. Relationship between the tumor necrosis factor receptor II (TNF-RII) gene polymorphism and sTNF-RII plasma levels in healthy controls and in rheumatoid arthritis. Hum Immunol. 2004; 65(12):1420-6.
23. Spoettl T, Hausmann M, Klebl F, Dirmeier A, Klump B, Hoffmann J, Herfarth H, Timmer A, Rogler G. Serum soluble TNF receptor I and II levels correlate with disease activity in IBD patients. Inflamm Bowel Dis. 2007; 13(6):727-32.
24. Takahashi J, Ebara S, Kamimura M, Kinoshita T, Misawa H, Shimogata M, Tozuka M, Takaoka K. Pro-inflammatory and anti-inflammatory cytokine increases after spinal instrumentation surgery. J Spinal Disord Tech. 2002; 15(4):294-300.
25. Cox FA, Stiller-Winkler R, Hadnagy W, Ranft U, Idel H. Soluble tumor necrosis factor receptor (sTNF RII) in sera of children and traffic-derived particulate air pollution. Zentralbl Hyg Umweltmed. 1999; 202(6):489-500.
26. Jones SA, Rose-John S. The role of soluble receptors in cytokine biology: the agonistic properties of the sIL-6R/IL-6 complex. Biochim Biophys Acta. 2002; 1592(3):251-63.
27. Shariat SF, Chromecki TF, Hoefer J, Barbieri CE, Scherr DS, Karakiewicz PI, Roehrborn CG, Montorsi F, Culig Z, Cavarretta IT. Soluble gp130 regulates prostate cancer invasion and progression in an interleukin-6 dependent andindependent manner. J Urol. 2011; 186(5):2107-1457.
28. Kristiansen M, Graversen JH, Jacobsen C, Sonne O, Hoffman HJ, Law SK, Moestrup SK. Identification of the haemoglobin scavenger receptor. Nature 2001; 409 (6817):198-201
29. Högger P, Sorg C. Soluble CD163 inhibits phorbol ester-induced lymphocyte proliferation. Biochem Biophys Res Commun. 2001; 288(4):841-3.
30. Levy AP, Purushothaman KR, Levy NS, Purushothaman M, Strauss M, Asleh R, Marsh S, Cohen O, Moestrup SK, Moller HJ, Zias EA, Benhayon D, Fuster V, Moreno PR. Downregulation of the hemoglobin scavenger receptor in individuals with diabetes and the Hp 2-2 genotype: implications for the response to intraplaque hemorrhage and plaque vulnerability. Circ Res. 2007; 101(1):106-10.
31. Fabriek BO, Møller HJ, Vloet RP, van Winsen LM, Hanemaaijer R, Teunissen CE, Uitdehaag BM, van den Berg TK, Dijkstra CD. Proteolytic shedding of the macrophage scavenger receptor CD163 in multiple sclerosis. J Neuroimmunol. 2007 ;187(1-2):179-86.
32. Burdo T H, Janet Lo, Suhny Abbara, Jeffery Wei, Michelle E DeLelys, Fred Preffer, Eric s Rosenberg, Kenneth C. Williams, and Steven Grinspoon, Soluble CD163, a novel marker of activated macrophages, is elevated and associated with noncalcified coronary plaque in HIV-infected patients, The Journal of Infection Diseases 2011; 204:1227-36
33. Møller HJ, Grønbaek H, Schiødt FV, Holland-Fischer P, Schilsky M, Munoz S, Hassanein T, Lee WM; U.S. Acute Liver Failure Study Group. Soluble CD163 from activated macrophages predicts mortality in acute liver failure. J Hepatol. 2007; 47(5):671-6.
34. Melmed RN, Taylor JM, Detels R, Bozorgmehri M, Fahey JL. Serum neopterin changes in HIV-infected subjects: indicator of significant pathology, CD4 T cell changes, and the development of AIDS. J Acquir Immune Defic Syndr.1989; 2(1):70-6.
35. Fahey JL, Taylor JM, Detels R, Hofmann B, Melmed R, Nishanian P, Giorgi JV.

The prognostic value of cellular and serologic markers in infection with human Immunodeficiency virus type 1. N Engl J Med. 1990 ;322(3):166-72.
